# Prognostic visualization model for primary pulmonary sarcoma: a SEER-based study

**DOI:** 10.1038/s41598-023-45058-7

**Published:** 2023-10-18

**Authors:** Qian Huang, Wenqiang Li, Xiaoyu He, Qian He, Qun Lai, Quan Yuan, Zhiping Deng

**Affiliations:** 1 Sichuan North Medical College, Nanchong, 637000 Sichuan Province China; 2 Zigong First People’s Hospital, Zigong City, 643000 Sichuan Province China; 3 West China Second Hospital of Sichuan University, Chengdu, 610044 Sichuan Province China; 4 The First Hospital of Jilin University, Changchun, 130021 Jilin Province China

**Keywords:** Cancer, Medical research

## Abstract

Primary pulmonary sarcoma (PPS) is a rare and poor prognostic malignancy that results from current clinical studies are lacking. Our study aimed to investigate the prognostic factors of PPS and to construct a predictive nomogram that predict the overall survival (OS) rate. We extracted data on patients diagnosed with PPS from 2010 to 2019 in the SEER database. A total of 169 patients were included after screening by inclusion and exclusion criteria. Univariate and multivariate COX regression analyses showed that age, pathological grade, liver metastasis, surgical intervention, and chemotherapy influenced the prognosis. We constructed the prediction model nomogram based on these factors. Moreover, the results of the internal and external ROC curves, calibration curves, and DCA plots confirmed that the model has good discrimination, accuracy, and clinical practice efficacy. The present study is the first population-based study to explore the factors affecting the prognosis of PPS. We established a novel prognostic nomogram to predict the OS rate, which can help to make proper clinical decisions.

## Introduction

Primary pulmonary sarcoma (PPS) is a very rare malignant mesenchymal tumor that accounts for 0.013–0.4% of lung cancers^1,2^. The cumulative number of PPS cases in most healthcare institutions has been less than 50 for more than 20 years^1,3–5^. Histologically, PPS is usually categorized as malignant fibrous histiocytoma, smooth muscle sarcoma, rhabdomyosarcoma, synovial sarcoma, etc. The 5-year OS rate of PPS is lower than that of limb soft-tissue sarcoma, and the median survival (mOS) is 21 months^6^. The prognosis of PPS is affected by a variety of factors, and the most frequently discussed therapy method is the surgery, which is more often seen in primary pulmonary artery sarcoma^7^. In addition, the prognosis of PPS is influenced by age, gender, smoking history, tumor size, and radiotherapy, but these are more commonly seen in case reports respectively^8–11^.

Due to the low incidence and mostly disseminated nature of PPS, a better treatment system has not yet been established. So in the last decades any significant progress wasn’t got in survival prognosis. There are fewer clinical reports that focus on the effect of individual factors on the prognosis of PPS patients. Meanwhile, studies have found that PPS patients are mostly middle-aged, with an average age of around 50 years^1,3,4,12^. What is known is that along with the popularity of high-resolution CT, the number of rare tumors diagnosed in the lungs is increasing every year. Therefore, it is necessary to analyze the multifactorial prognosis. The SEER database contains multicenter data of oncology patients, which is rich and reliable. As a result, it can provide a large sample for research. Nomograms, with the fetures of visualization and quantification, are widely used as tools for prognostic evaluation of malignant tumors^13,14^. Based on the above, we investigated the clinical features and prognostic factors of PPS using the SEER database, and constructed a novel nomogram prediction model to predict the OS rate. Meantime, we performed multidimensional validation to fully confirm the good predictive efficacy of the model, which will provide objective and scientific guidance for clinical decision-making.

## Materials and methods

### Source

The SEER database is an open-access cancer database covering 28% of the US population. We extracted data for PPS patients in “Incidence-SEER 17 Regs Custom Data (with additional treatment fields), Nov 2021 Sub(2000–2019)” from 2010 to 2019.

### Patients

**Figure 1:**
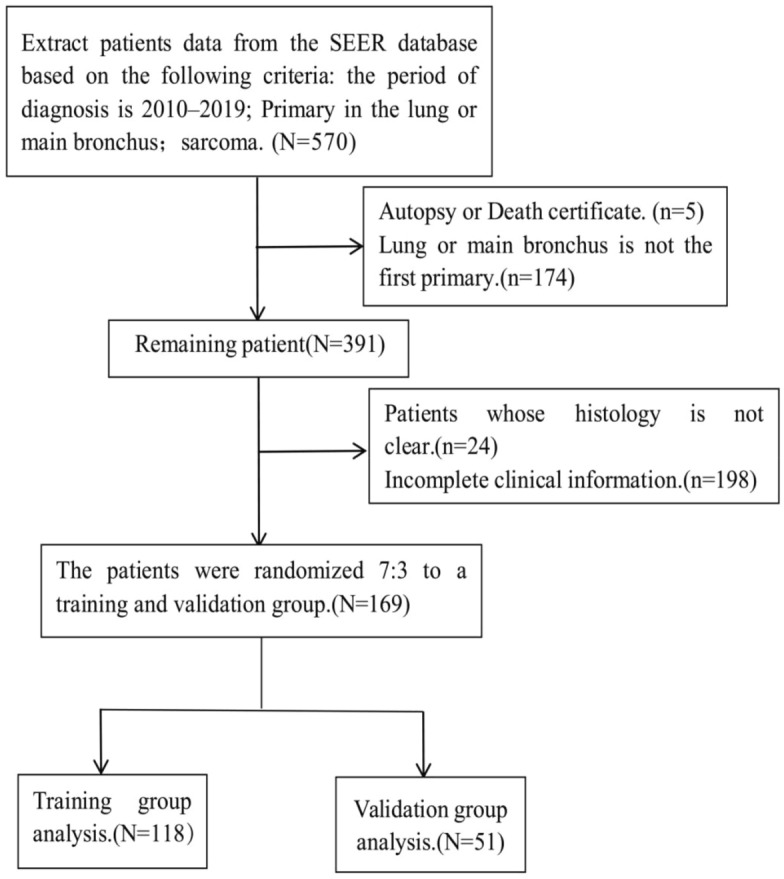
The patients screening process.

Patient screening criteria were as follows: (1) Inclusion criteria: first primary in lung and bronchus; ICD-O-3 Hist/Behav (8800-8805/3, 8810/3, 8811/3, 8840/3, 8850/3, 8851/3, 8854/3, 8890/3, 8891/3, 8894/3, 8896/3 8900-8902/3, 8910/3, 8912/3, 8920/3, 8921/3, 8933/3, 8936/3, 9040-9044/3, 9260/3); pathological diagnosis. (2) Exclusion criteria: patients diagnosed by autopsy or death certificate; patients with incomplete required clinical information.OS was the primary study endpoint. The following information was collected: demographic variables, including age, sex, race, and marital status; clinicopathological information, including the year of diagnosis, survival time, pulmonary metastases, bone metastases, brain metastases, liver metastases, surgery, radiotherapy, and chemotherapy. Finally, a total of 169 patients with PPS were included in this study and randomized into the development and validation groups in a 7:3 ratio (Fig. 1). We constructed a prediction model with patient data from the development training group, and then validated the model internally with patients in the development training group and externally with patients in the validation group.

### Statistical analysis

Statistical analysis was performed using R software (version 4.2.1), and *P* < 0.05 (bilateral) was considered statistically significant. The chi-square test was used to compare the distribution of variables in the modeling and validation groups. Then, univariate and multivariate COX regression analyses were used to stepwise identify independent prognostic factors. We built a novel predictive nomogram based on the independent prognostic factors to predict the OS rate of PPS patients at 1, 2 and 3 years, and constructed survival curves by Kaplan–Meier analysis to specify the median survival (mOS) of the relevant factors. In addition, time-dependent receiver operating characteristic (ROC) curves for 1, 2, and 3 years of nomogram were generated and the corresponding time-dependent area under the curve (AUC) was applied to discriminate. AUC < 0.6 was considered as poor discriminatory ability, 0.6–0.75 as fair, and > 0.75 as good. In addition, calibration curves and decision curve analysis (DCA) were plotted to assess the nomogram.

### Ethics approval

We signed the SEER Study Data Agreement to access SEER information using reference number 20225-Nov2021. We performed study methods following approved guidelines to access data provided by the SEER database. The Office of Human Research Protections considers data analysis to be non-human subjects studied by the U.S. Department of Health and Human Services because they are publicly available and de-identified. Therefore, it does not require any approval from the Institutional Review Board.

## Results

### Baseline characteristics of PPS patients

The SEER database was extracted from 570 patients with PPS, which included 98 cases of synovial sarcoma. It was the most common pathological type of sarcoma, accounting for approximately 17.19% of all PPS. After screening by inclusion and exclusion criteria, a total of 169 patients with PPS were finally included in this study, with an mOS of 7 months. PPS was mostly seen in older men with higher pathological grading. Distant metastases occurred in about 28.4% of them, with pulmonary metastases being the most common, accounting for 10.7% of all and 37.5% of distant metastases. Nearly half of the patients underwent surgical intervention and about one-third received chemotherapy and radiotherapy. All patients were randomly divided in a 7:3 ratio into a development training group of 118 (70%) and a validation group of 51 (30%), and the results of chi-square test showed that all variables were not statistically different between the two groups (Table 1).

**Table 1:** Basic characteristics of PPS patients.

	Development training group	Validation group	Overall	χ^2^	*P*
	(N = 118)	(N = 51)	(N = 169)		
Age				0.014	0.904
< 65	55 (46.6%)	25 (49.0%)	80 (47.3%)		
≥ 65	63 (53.4%)	26 (51.0%)	89 (52.7%)		
Sex				1.152	0.283
Female	49 (41.5%)	16 (31.4%)	65 (38.5%)		
Male	69 (58.5%)	35 (68.6%)	104 (61.5%)		
Race				3.917	0.141
Black	6 (5.1%)	6 (11.8%)	12 (7.1%)		
Other	12 (10.2%)	2 (3.9%)	14 (8.3%)		
White	100 (84.7%)	43 (84.3%)	143 (84.6%)		
Marital				0.203	0.653
Married	61 (51.7%)	29 (56.9%)	90 (53.3%)		
Other	57 (48.3%)	22 (43.1%)	79 (46.7%)		
Grade				0.088	0.767
I-II	20 (16.9%)	7 (13.7%)	27 (16.0%)		
III-IV	98 (83.1%)	44 (86.3%)	142 (84.0%)		
Metastasis					
Lung				< 0.001	1.000
No/Unknown	105 (89.0%)	46 (90.2%)	151 (89.3%)		
Yes	13 (11.0%)	5 (9.8%)	18 (10.7%)		
Bone				< 0.001	0.988
No/Unknown	107 (90.7%)	47 (92.2%)	154 (91.1%)		
Yes	11 (9.3%)	4 (7.8%)	15 (8.9%)		
Liver				< 0.001	1.000
No/Unknown	115 (97.5%)	49 (96.1%)	164 (97.0%)		
Yes	3 (2.5%)	2 (3.9%)	5 (3.0%)		
Brain				1.162	0.281
No/Unknown	109 (92.4%)	50 (98.0%)	159 (94.1%)		
Yes	9 (7.6%)	1 (2.0%)	10 (5.9%)		
Surgery				0.046	0.829
No	61 (51.7%)	28 (54.9%)	89 (52.7%)		
Yes	57 (48.3%)	23 (45.1%)	80 (47.3%)		
Radiation				2.971	0.085
No/Unknown	75 (63.6%)	40 (78.4%)	115 (68.0%)		
Yes	43 (36.4%)	11 (21.6%)	54 (32.0%)		
Chemotherapy				0.060	0.807
No/Unknown	78 (66.1%)	32 (62.7%)	110 (65.1%)		
Yes	40 (33.9%)	19 (37.3%)	59 (34.9%)		

**Grade**: I, Well differentiated; II, Moderately differentiated; III, Poorly differentiated; IV, Undifferentiated. ***P***: Values calculated by chi-square test.

### Prognostic factors and survival curves

**Figure 2:**
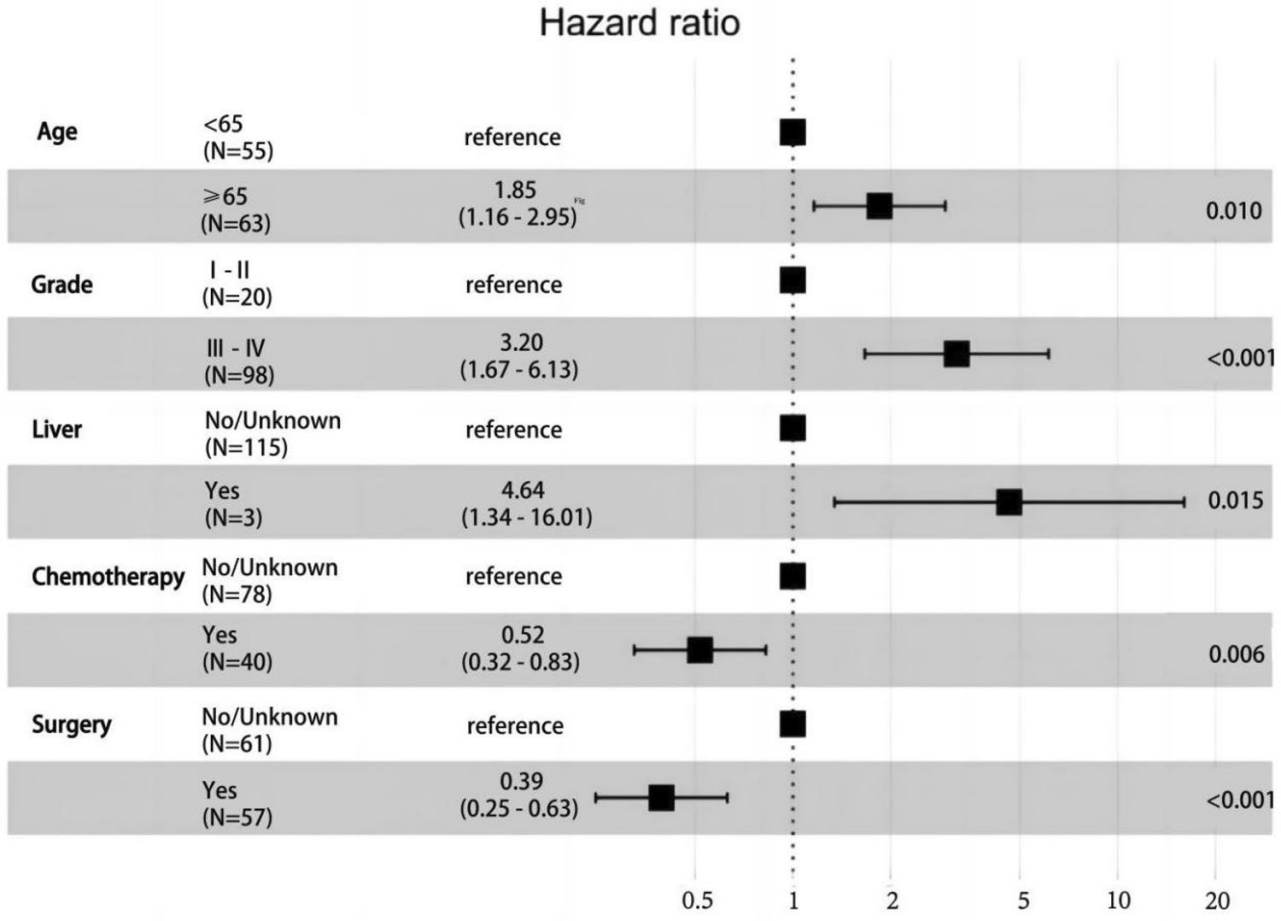
A forest plot of PPS independent prognostic factors.

**Figure 3:**
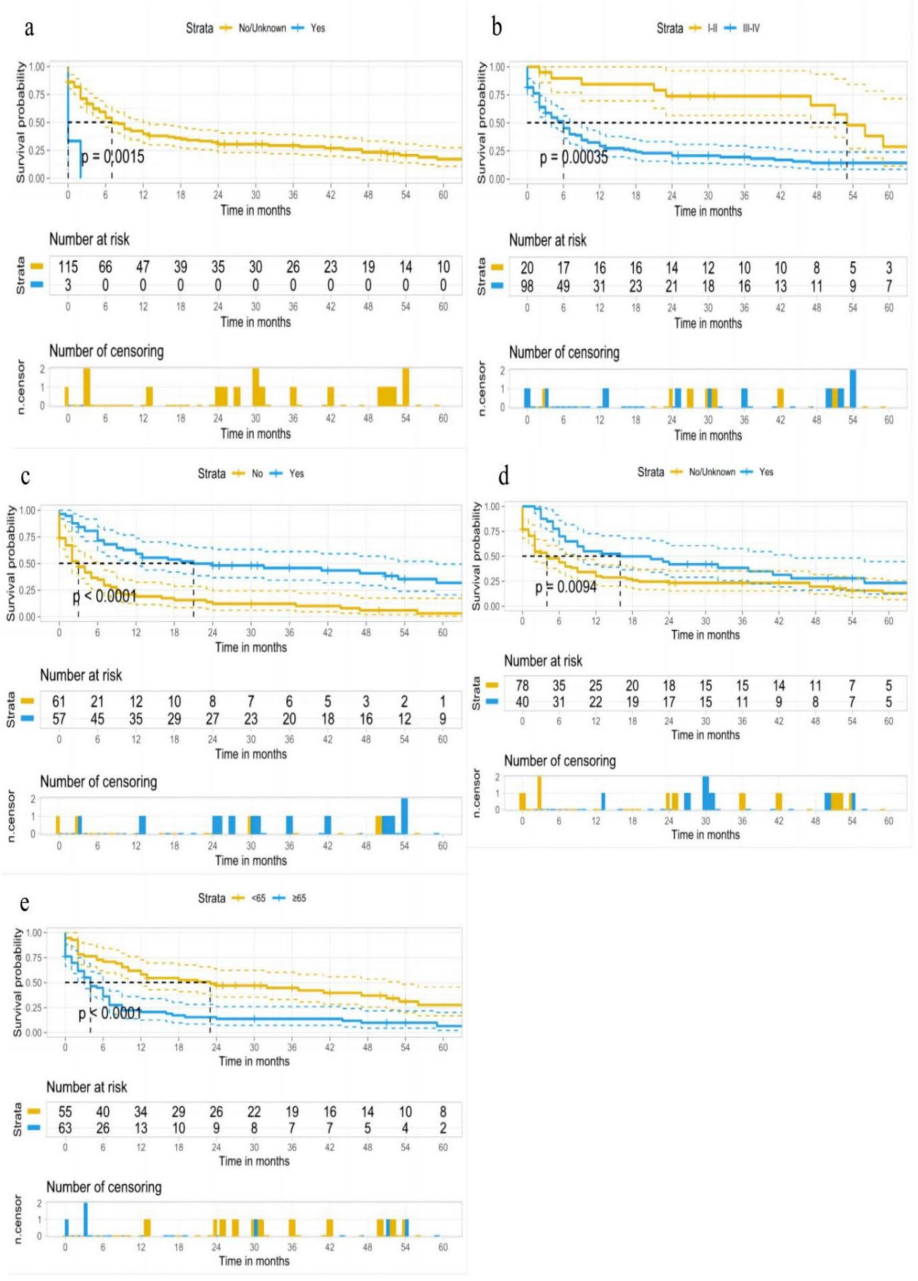
(**a**) OS by liver metastasis. (**b**) OS by pathological grading. (**c**) OS by Surgery. (**d**) OS by Chemotherapy. (**e**) OS by Age of diagnosis.

We included age, gender, race, marriage, pathological grade, distant metastasis (lung, liver, brain, bone), surgery, radiotherapy, and chemotherapy variables in the development training group for univariate COX regression analysis, and the results showed that age, pathological grade, liver metastasis, surgery, and chemotherapy were statistically significant (*P* < 0.05). We subjected the above statistically significant factors to multivariate COX regression analysis, and the results showed that all the above factors were statistically significant (Table 2). This indicated that age, pathological grade, liver metastasis, surgery and chemotherapy are independent factors affecting the prognosis of PPS. The forest plot visualizes the magnitude of each independent factor and ranks them (in order of liver metastasis, pathologic grade, chemotherapy, and age). Patients with PPS who were ≥ 65 years old, had liver metastases, and had higher pathological grading had a poorer prognosis, and those who received surgical and chemotherapeutic interventions improved their prognosis (Fig. 2). Then, we plotted survival curves for the above factors. The results showed that those who developed liver metastases had a poorer prognosis than those who did not, and none had an OS of 1 year (Fig. 3a). Those with high pathological grading had a worse prognosis than those with low grading (mOS: 6 months vs. 53 months, 5 year OS rate: 12.5% vs. 28%) (Fig. 3b). Those with surgical intervention had a better prognosis than those without surgery (mOS: 21 months vs. 3 months), with a 5 year OS rate of approximately 33% (Fig. 3c). Chemotherapy had a better prognosis than those without chemotherapy (mOS: 16 months vs. 4 months, 5-year OS rate: 25% vs. 12.5%) (Fig. 3d), and those ≥ 65 years had a worse prognosis than those < 65 (mOS: 4 months vs. 23 months, 5 year OS rate: 6% vs. 25%) (Fig. 3e).

**Table 2:** Prognostic factors for patients with PPS.

	Univariate analysis		Multivariate analysis	
	HR(95%CI)	*P*^*$*^	HR(95%CI)	*P*^*^*^
Age				
< 65	Reference		Reference	
≥ 65	2.443(1.604–3.722)	< 0.001	1.850(1.162–2.947)	0.010
Sex				
Female	Reference			
Male	0.914(0.607–1.375)	0.665		
Race				
Black	Reference			
Other	1.024(0.348–3.012)	0.966		
White	0.991(0.401–2.453)	0.985		
Marital				
Married	Reference			
Other	1.211(0.807–1.816)	0.355		
Grade				
I-II	Reference		Reference	
III-IV	3.012(1.597–5.682)	< 0.001	3.196(1.666–6.131)	< 0.001
Metastasis				
Lung				
No/Unknown	Reference			
Yes	1.51 (0.781–2.921)	0.221		
Bone				
No/Unknown	Reference			
Yes	1.717(0.886–3.329)	0.109		
Liver				
No/Unknown	Reference		Reference	
Yes	7.118(2.153–23.530)	0.001	4.638(1.343–16.015)	0.015
Brain				
No/Unknown	Reference			
Yes	2.000(0.991–4.034)	0.053		
Surgery				
No	Reference		Reference	
Yes	0.329( 0.215–0.504)	< 0.001	0.394(0.247–0.628)	< 0.001
Radiation				
No/Unknown	Reference			
Yes	0.871(0.574–1.322)	0.517		
Chemotherapy				
No/Unknown	Reference		Reference	
Yes	0.553(0.357–0.859)	0.008	0.518(0.325–0.826)	0.006

**Grade**: I, Well differentiated; II, Moderately differentiated; III, Poorly differentiated; IV, Undifferentiated. *P*^*$*^: Values calculated by univariate COX regression analysis. *P*^*^*^: Values calculated by multivariate COX regression analysis.

### Development training and validation of the nomogram

**Figure 4:**
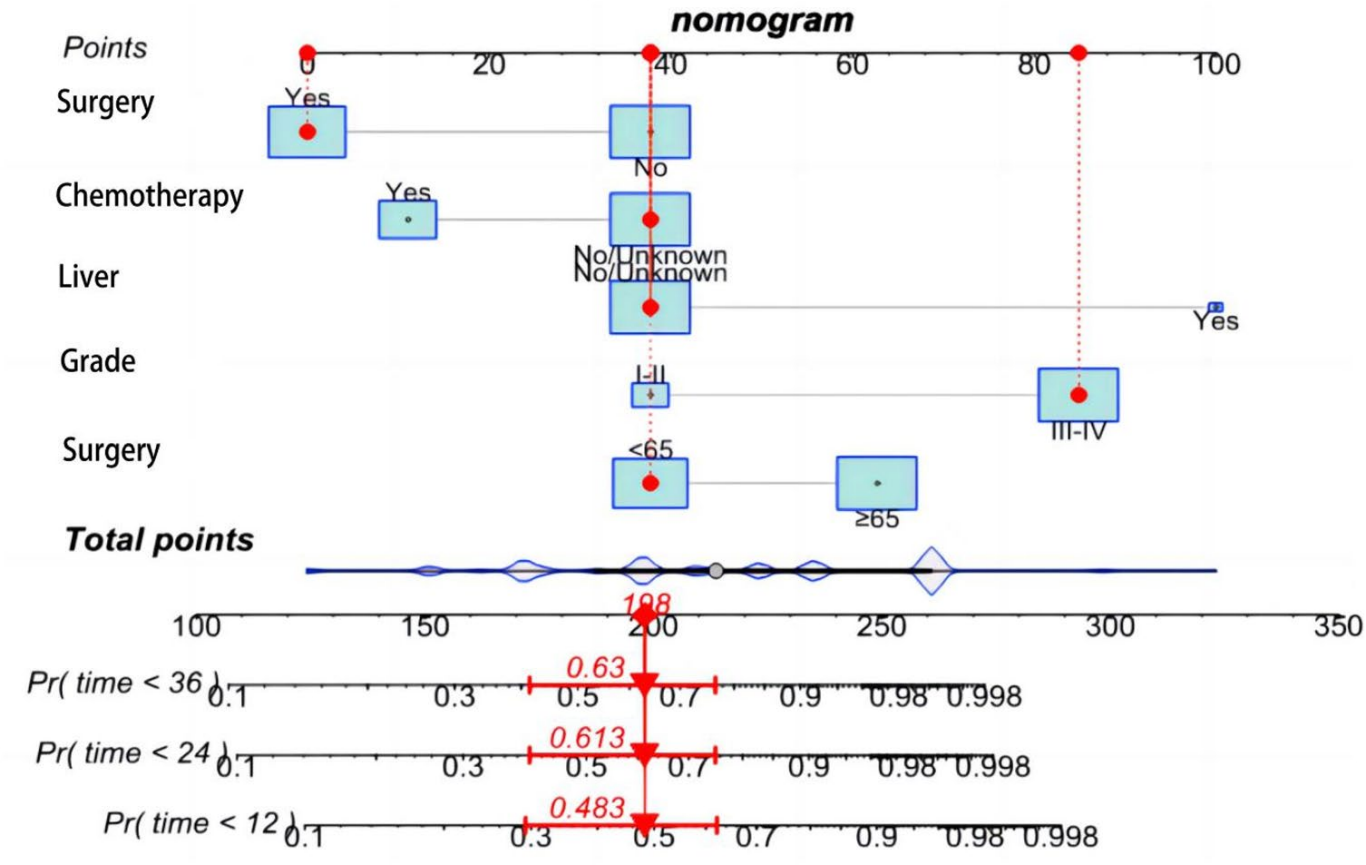
A prognostic nomogram to predict the 1, 2, and 3 years OS in PPS patients.

**Figure 5:**
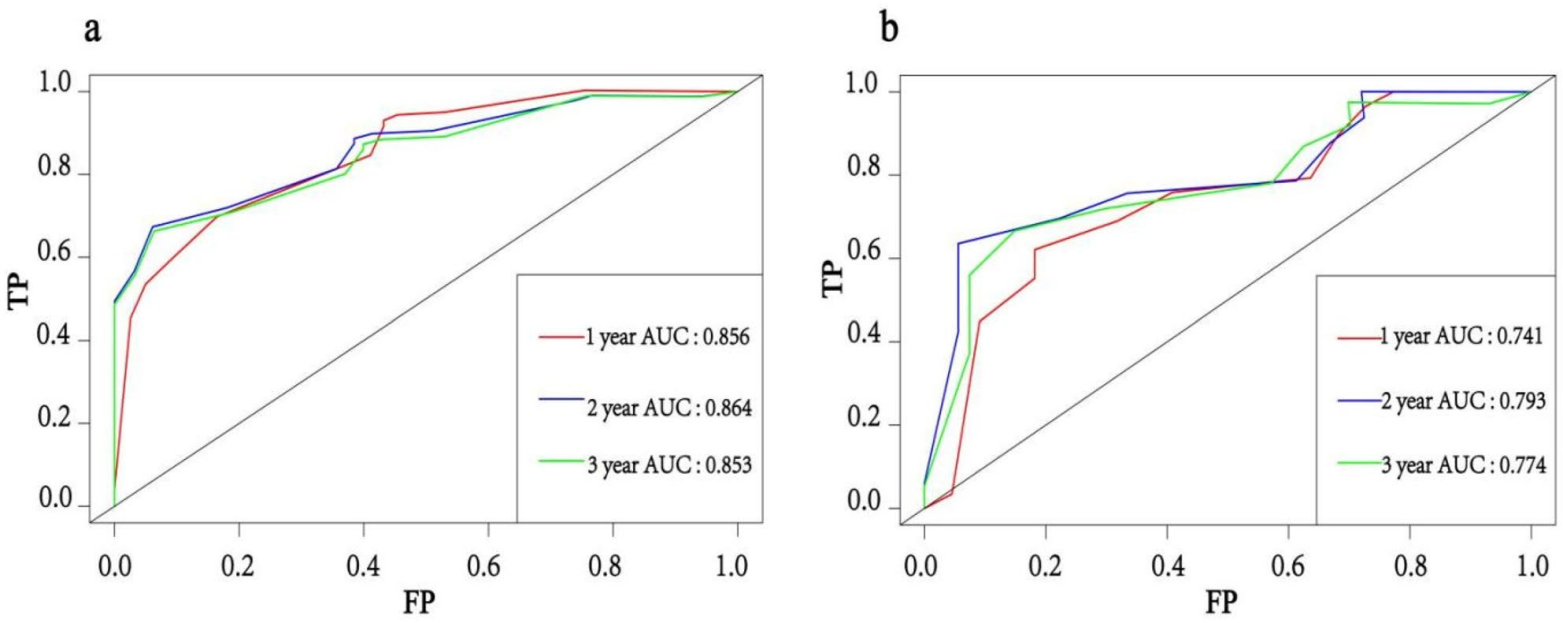
Time-dependent ROC curve analysis of the nomogram for the 1, 2, and 3 years in the training set (**a**) and the validation set (**b**).

**Figure 6:**
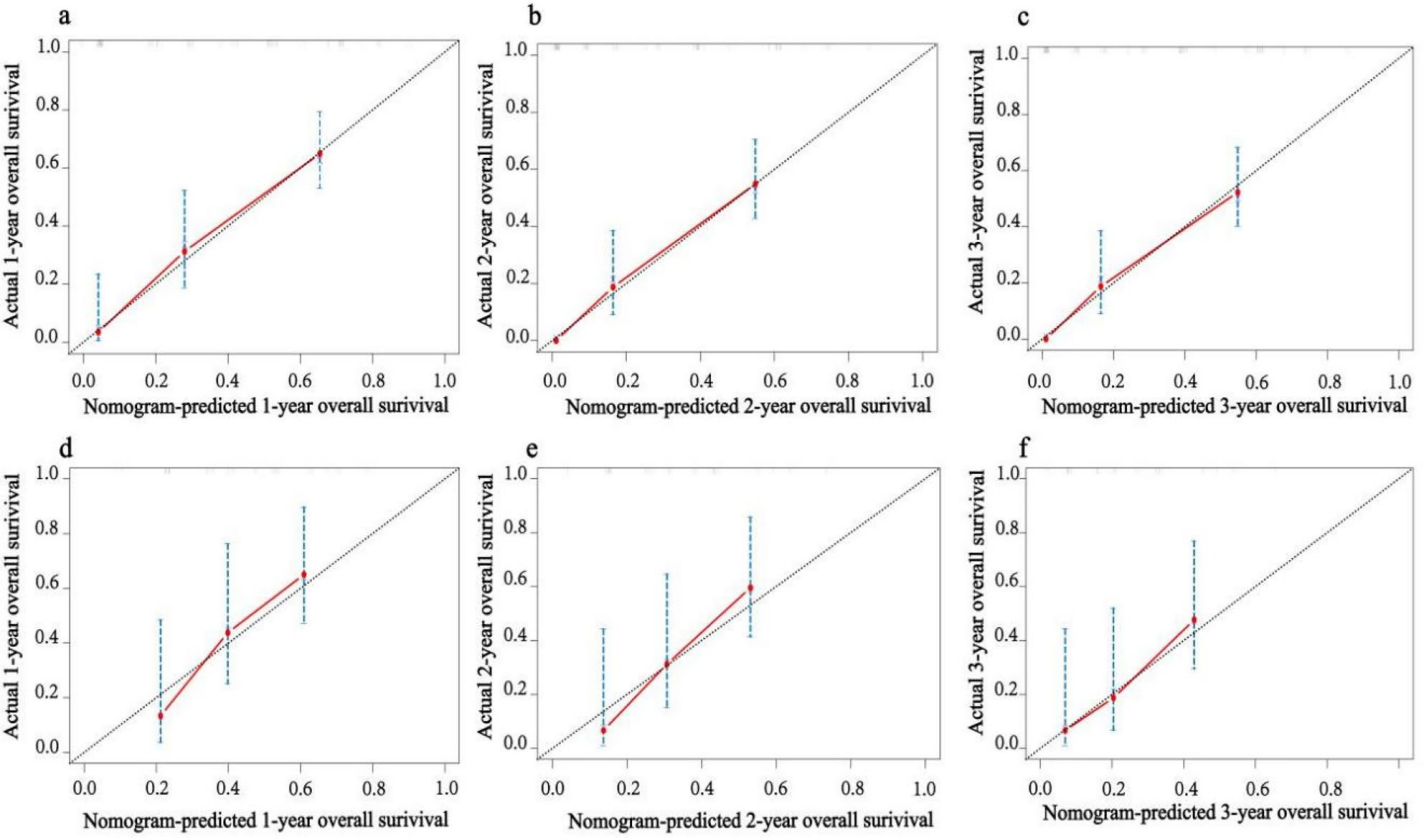
The calibration curves for predictions of overall survival in the training set (**a**-**c**) and in the validation set (**d**-**f**) at 1, 2, and 3 years.

**Figure 7:**
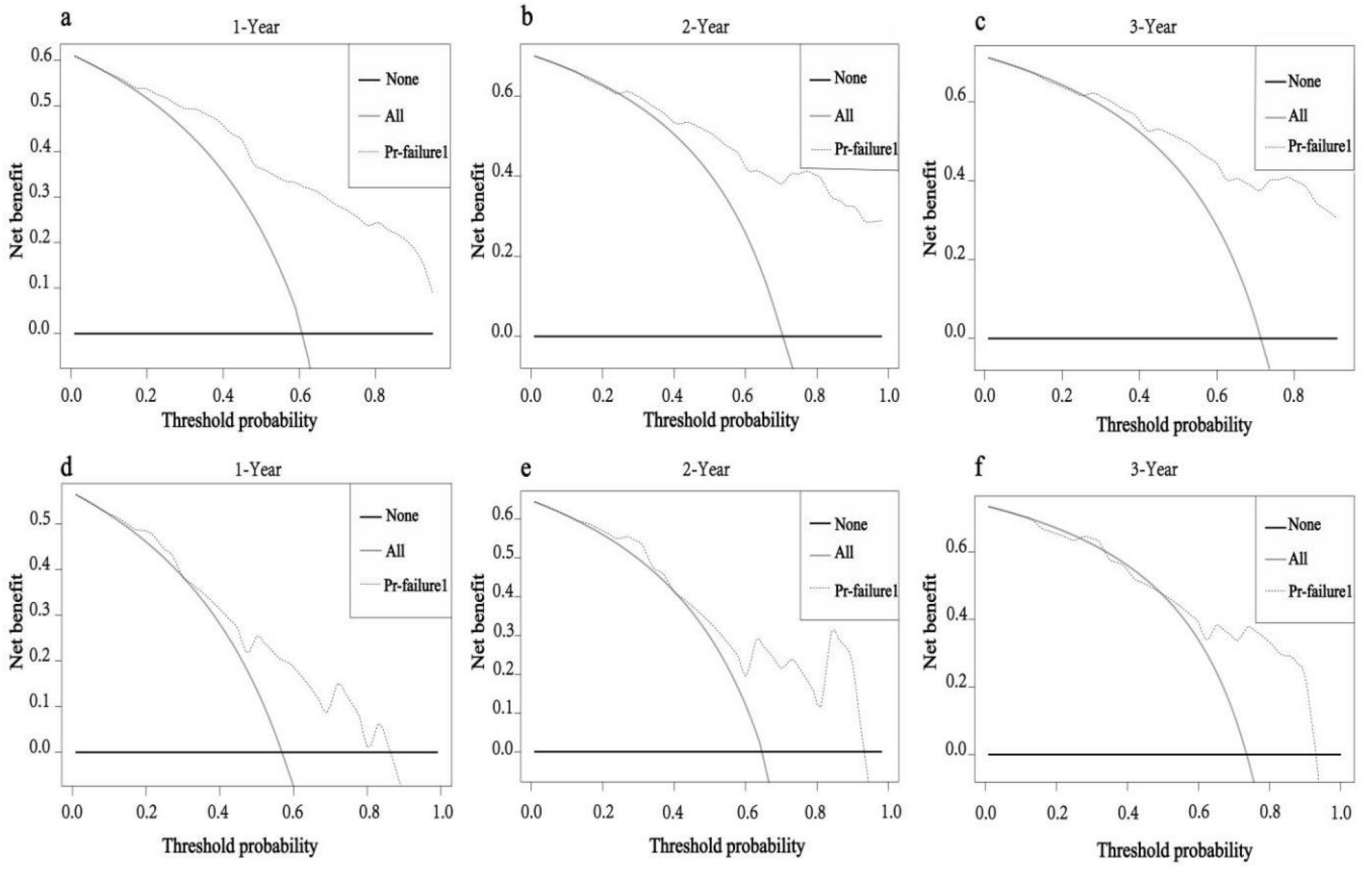
The decision curve analysis of the nomogram in the training set (**a**-**c**) and in the validation set (**d**-**f**) at 1, 2, and 3 years.

Based on the independent prognostic factors obtained above, we constructed a novel prognostic nomogram to visually calculate the OS rates of PPS patients at 1, 2, and 3 years (Fig. 4). Then, the differentiation ability of the Nomogram was assessed by plotting ROC curves and calculating the corresponding AUC values. The AUC values for the development training group were 0.856, 0.864, and 0.853 at 1, 2, and 3 years, respectively (Fig. 5a), and the AUC values for the validation group were 0.741, 0.793, and 0.741, respectively (Fig. 5b), indicating that the model has good discriminatory power. The internal (Fig. 6a–c) and external (Fig. 6d–f) calibration plots showed that the 1-, 2-, and 3-year OS rates predicted by the column line plots were similar to the actual 1-, 2-, and 3-year OS rates with a high degree of consistency. In addition, the internal (Fig. 7a–c) and external (Fig. 7d–f) DCA plots also indicated that the model has clinical practice efficacy.

## Discussion

Sarcoma is a malignant tumor originating from mesenchymal tissues such as fibrous tissue, fat, muscle, blood vessels and lymphatic vessels, bone, and cartilage tissue. We extracted more than a dozen pathological types of sarcomas commonly originating from the lung in the last decade from the SEER database, such as fibrosarcoma, synovial sarcoma, liposarcoma, smooth muscle sarcoma, rhabdomyosarcoma, and angiosarcoma, etc. To our statistical analysis, synovial sarcoma was the most common pathological type, accounting for 17.19%, which was inconsistent with previous studies. Some studies reported that smooth muscle sarcoma was the most commonly reported type of PPS^15,16^. The sudy of Petrov et al. in 48 cases of PPS was fibrosarcoma, which accounted for 37.5%^4^. We considered more abundant and larger samples in the SEER database.PPS is a very rare malignancy with an inferior poor prognosis. And most of the studies on PPS were case reports or were single-center case series, and large samples are scarce. No effective treatment has been found so far. Evaluation of prognostic factors will help clinicians to make scientific and effective clinical decisions. Therefore, we explored the independent factors affecting the prognosis and found that liver metastasis, pathological grade, surgery, age, and chemotherapy have a significant effect on its prognosis. And we also developed a novel predictive nomogram, which can be used to visually personalize the OS rates at 1, 2, and 3 years. In Fig. 4, a patient with PPS < 65 years old, with pathological grade III-IV, without liver metastases and treated only with surgery, had a total of 198 points calculated by the model, and the corresponding 1-year, 2-year, and 3-year OS rates were 51.7%, 38.7%, and 37%, respectively. If the patient also received chemotherapy, the total number of points calculated by the model would be approximately 168, with corresponding 1-year, 2-year, and 3-year OS rates of 72%, 62.5%, and 62%, respectively. Thus, postoperative chemotherapy for PPS significantly improved OS. In addition, we validated the model, the results showed that it has good discrimination, predictive accuracy, and also favorable clinical practice utility, which can be used to clinically guide clinical decision-making.

According to the model we constructed, the main treatments for PPS are surgery, chemotherapy, and radiotherapy, consistent with other common solid malignancies. Complete tumor resection is the widely accepted treatment for PPS. Some endobronchial sarcomas can be treated with conservative pneumonectomy, including carina excision^18,19^. Previous studies have shown that the resection rate for PPS is 50–100%^1,3,4,14,19^, while our results was slightly lower, with approximately 47.3% undergoing surgical treatment. PPS is more aggressive and has a lower survival rate compared to other malignancies originating from the lung, with a 3-year postoperative survival rate of 17–50%^6,12,15,16^. Bacha et al.^17^ showed a 5-year postoperative OS rate of 50% for PPS, while the 5-year postoperative OS rate for PPS in the present study was 33%, lower than it. It may be because most of the PPS patients included in this study had a high pathological grade. Sarcomas with high-grade malignancies have the worse prognosis than lower grades^15^. Similar findings were found in our study, where patients with pathological grade III–IV had a significantly lower mOS than grade I–II (6 months vs. 53 months). Moreover, Régnard et al.^1^ and Petrov et al.^4^ studies confirmed that those with the earlier TNM stages in PPS patients favored survival. Several earlier studies also showed^1,15,19^ that patients who underwent complete resection had significantly higher OS rates compared to inoperability or positive surgical margins. Therefore, early complete tumor resection is particularly important to improve OS rates in PPS^3,4^. However, radiotherapy does not benefit patients in some cases. Spraker et al.^6^ showed that was no statistical difference in OS between patients who received surgery and radiotherapy and those who received surgery alone. In our study, we also did not find that radiotherapy improved the OS rate of PPS.

More interestingly, pulmonary sarcomatoid carcinoma (PSC) is a group of poorly differentiated non-small cell lung cancer (NSCLC) containing a sarcomatoid component, including giant cell carcinoma, spindle cell carcinoma, pleomorphic carcinoma, and carcinosarcoma. In some cases, PSC is difficult to distinguish from pulmonary sarcoma (PS), and PS has a better prognosis than PSC^20,21^ and worse than NSCLC^21^. The OS rate of PPS we showed was also better compared to that of PSC shown in previous studies^22^. Moreover, chemotherapy does not improve the OS rate of PSC^22–25^, whereas our study showed that chemotherapy improves the OS rate of PPS. Therefore, we must correctly identify PSC with PPS to make appropriate treatment. Age and liver metastasis were independent factors affecting the prognosis of PPS in our study. Elderly people (≥ 65 years) and those who developed liver metastases had a poor prognosis, none of whom survived more than one year. In addition, pulmonary, bone, and brain metastases had no significant effect on the prognosis of PPS. Although some the prognostic factors are to be expected. in our study, We further quantified them and clarified the magnitude of the weighting of each factor. To our knowledge, this is the largest sample size study with real-world data on PPS prognosis. Consequently, our research is great significance and clinical guiding value.

Some limitations are still in our study. On the one hand, the SEER database does not include information on specific regimens of chemotherapy, radiotherapy, and surgery, so we cannot discuss the pros and cons of specific treatment regimens on the prognosis of PPS. On the other hand, due to the limitation of retrospective study and the long time span, our conclusions may be biased. However, the SEER database is still in the process of being updated, and we believe that these issues will be resolved soon.

## Data Availability

The datasets for this study can be found in the SEER*Stat Software (cancer.gov). The original contributions presented in the study are included in the article.

## References

[1] Régnard JF, Icard P, Guibert L, de Montpreville VT, Magdeleinat P, Levasseur P (1999). Prognostic factors and results after surgical treatment of primary sarcomas of the lung. Ann. Thorac. Surg..

[2] Guccion JG, Rosen SH (1972). Bronchopulmonary leiomyosarcoma and fibrosarcoma. A study of 32 cases and review of the literature. Cancer.

[3] Porte HL, Metois DG, Leroy X, Conti M, Gosselin B, Wurtz A (2000). Surgical treatment of primary sarcoma of the lung. Eur. J. Cardiothorac. Surg..

[4] Petrov DB, Vlassov VI, Kalaydjiev GT, Plochev MA, Obretenov ED, Stanoev VI, Danon SE (2003). Primary pulmonary sarcomas and carcinosarcomas–postoperative results and comparative survival analysis. Eur. J. Cardiothorac. Surg..

[5] Robinson LA, Babacan NA, Tanvetyanon T, Henderson-Jackson E, Bui MM, Druta M (2021). Results of treating primary pulmonary sarcomas and pulmonary carcinosarcomas. J. Thorac. Cardiovasc. Surg..

[6] Spraker MB, Bair E, Bair R, Connell PP, Mahmood U, Koshy M (2013). An analysis of patient characteristics and clinical outcomes in primary pulmonary sarcoma. J. Thorac. Oncol..

[7] Yin K, Zhang Z, Luo R, Ji Y, Zheng D, Lin Y, Wang C (2018). Clinical features and surgical outcomes of pulmonary artery sarcoma. J. Thorac. Cardiovasc. Surg..

[8] Berzenji L, Van Schil PE (2021). Commentary: Primary pulmonary sarcomas and pulmonary carcinosarcomas, challenging and enigmatic, but treatable!. J. Thorac. Cardiovasc. Surg..

[9] Yeo SG (2017). Primary lung sarcoma treated with stereotactic ablative radiotherapy: A case report. Onco Targets Ther..

[10] Collaud S, Stork T, Schildhaus HU, Pöttgen C, Plönes T, Valdivia D, Zaatar M, Dirksen U, Bauer S, Aigner C (2020). Multimodality treatment including surgery for primary pulmonary sarcoma: Size does matter. J. Surg. Oncol..

[11] Tavora F, Miettinen M, Fanburg-Smith J, Franks TJ, Burke A (2008). Pulmonary artery sarcoma: A histologic and follow-up study with emphasis on a subset of low-grade myofibroblastic sarcomas with a good long-term follow-up. Am. J. Surg. Pathol..

[12] Etienne-Mastroianni B, Falchero L, Chalabreysse L, Loire R, Ranchère D, Souquet PJ, Cordier JF (2002). Primary sarcomas of the lung: A clinicopathologic study of 12 cases. Lung Cancer..

[13] Balachandran VP, Gonen M, Smith JJ, DeMatteo RP (2015). Nomograms in oncology: More than meets the eye. Lancet Oncol..

[14] Jia J, Chen W (2021). Role of radiation therapy in primary tonsil large B cell lymphoma: A SEER-based analysis. Radiat. Oncol..

[15] Janssen JP, Mulder JJ, Wagenaar SS, Elbers HR, van den Bosch JM (1994). Primary sarcoma of the lung: A clinical study with long-term follow-up. Ann. Thorac. Surg..

[16] Attanoos RL, Appleton MA, Gibbs AR (1996). Primary sarcomas of the lung: A clinicopathological and immunohistochemical study of 14 cases. Histopathology.

[17] Bacha EA, Wright CD, Grillo HC, Wain JC, Moncure A, Keel SB, Donahue DM, Mathisen DJ (1999). Surgical treatment of primary pulmonary sarcomas. Eur. J. Cardiothorac. Surg..

[18] Muscolino G, Bedini AV, Buffa PF (2000). Leiomyosarcoma of the bronchus: Report of two cases of resection with long-term follow-up. J. Thorac. Cardiovasc. Surg..

[19] Magné N, Porsin B, Pivot X, Tchiknavorian X, Marcy PY, Foa C, Otto J, Schneider M, Thyss A (2001). Primary lung sarcomas: Long survivors obtained with iterative complete surgery. Lung Cancer.

[20] Steuer CE, Behera M, Liu Y, Fu C, Gillespie TW, Saba NF, Shin DM, Pillai RN, Pakkala S, Owonikoko TK, Khuri FR, Ramalingam SS (2017). Pulmonary sarcomatoid carcinoma: An analysis of the national cancer data base. Clin. Lung Cancer.

[21] Li AX, Resio BJ, Canavan ME, Papageorge M, Boffa DJ, Blasberg JD (2021). Outcomes of surgically managed primary lung sarcomas: A national cancer database analysis. J. Thorac. Dis..

[22] Liang L, Liu Z, Wang C, Xie S (2022). Adjuvant chemotherapy is not a decisive factor in improving the overall survival of pulmonary sarcoma: A population-based study. Front. Oncol..

[23] Vieira T, Girard N, Ung M, Monnet I, Cazes A, Bonnette P, Duruisseaux M, Mazieres J, Antoine M, Cadranel J, Wislez M (2013). Efficacy of first-line chemotherapy in patients with advanced lung sarcomatoid carcinoma. J. Thorac. Oncol..

[24] Hong JY, Choi MK, Uhm JE, Park MJ, Lee J, Park YH, Ahn JS, Park K, Han JH, Ahn MJ (2009). The role of palliative chemotherapy for advanced pulmonary pleomorphic carcinoma. Med. Oncol..

[25] Lee J, Jung HA, Kim Y, Choi S, Han J, Choi YL, Lee SH, Ahn JS, Park K, Sun JM (2018). Efficacy of mesna, doxorubicin, ifosfamide, and dacarbazine (MAID) in patients with advanced pulmonary pleomorphic carcinoma. Lung Cancer..

